# Cytokine release from alveolar macrophages exposed to ambient particulate matter: Heterogeneity in relation to size, city and season

**DOI:** 10.1186/1743-8977-2-4

**Published:** 2005-08-17

**Authors:** Ragna B Hetland, Flemming R Cassee, Marit Låg, Magne Refsnes, Erik Dybing, Per E Schwarze

**Affiliations:** 1 Division of Environmental Medicine, Norwegian Institute of Public Health, P.O. Box 4404 Nydalen, N-0403 Oslo, Norway; 2 Centre for Environmental Health Research, National Institute for Public Health and the Environment, P.O. Box 1, NL-3720 BA Bilthoven, the Netherlands

## Abstract

**Background:**

Several studies have demonstrated an association between exposure to ambient particulate matter (PM) and respiratory and cardiovascular diseases. Inflammation seems to play an important role in the observed health effects. However, the predominant particle component(s) that drives the inflammation is still not fully clarified. In this study representative coarse (2.5–10 μm) and fine (0.1–2.5 μm) particulate samples from a western, an eastern, a northern and a southern European city (Amsterdam, Lodz, Oslo and Rome) were collected during three seasons (spring, summer and winter). All fractions were investigated with respect to cytokine-inducing potential in primary macrophages isolated from rat lung. The results were related to the physical and chemical parameters of the samples in order to disclose possible connections between inflammatory potential and specific characteristics of the particles.

**Results:**

Compared on a gram-by gram basis, both site-specific and seasonal variations in the PM-induced cytokine responses were demonstrated. The samples collected in the eastern (Lodz) and southern (Rome) cities appeared to be the most potent. Seasonal variation was most obvious with the samples from Lodz, with the highest responses induced by the spring and summer samples. The site-specific or seasonal variation in cytokine release could not be attributed to variations in any of the chemical parameters. Coarse fractions from all cities were more potent to induce the inflammatory cytokines interleukin-6 and tumour necrosis factor-α than the corresponding fine fractions. Higher levels of specific elements such as iron and copper, some polycyclic aromatic hydrocarbons (PAHs) and endotoxin/lipopolysaccaride seemed to be prevalent in the coarse fractions. However, variations in the content of these components did not reflect the variation in cytokine release induced by the different coarse fractions. Addition of polymyxin B did not affect the particle-induced cytokine release, indicating that the variations in potency among the coarse fractions are not explained by endootoxin.

**Conclusion:**

The inflammatory potential of ambient PM demonstrated heterogeneity in relation to city and season. The coarse particle fractions were consistently more potent than the respective fine fractions. Though a higher level of some elements, PAH and endotoxin was found in the coarse fractions, the presence of specific components was not sufficient to explain all variations in PM-induced cytokine release.

## Background

Previous epidemiological studies in Europe have demonstrated a heterogeneity in respiratory and cardiovascular diseases after exposure to ambient particulate matter (PM) [1,2]. Inflammation plays a crucial part in the long-term development of lung diseases, and possibly also cardiovascular diseases induced by particles. Exacerbation of inflammatory responses also seems to be involved in the acute effects triggered by short-term exposure to particles [3-5].

The composition of PM is influenced by emissions from different sources such as traffic, industrial activities, residential heating and long distance transported air pollution. The PM may in certain areas contain considerable amounts of mineral particles generated by road surface abrasion, due to the use of studded tires during winter season. Occasionally, a significant part of the long distance transported PM also consists of mineral particles from the dry southern parts of Europe and Africa. Furthermore, temporal variations in source emissions and/or seasonal variations in temperature and other meteorological conditions may influence the composition of ambient PM. As a result, considerable site-specific and seasonal variations in the physical and chemical characteristics of particulate air pollution may occur.

Particle size is a critical parameter due to differential deposition in the respiratory tract, but also due to differential effects on the lung cells *per se*. Epidemiological data suggest that fine ambient particles may be more important than coarse in PM-associated mortality and adverse respiratory health effects [6-8]. Conversely, associations between PM and daily mortality in an environment in which PM is dominated by the coarse fraction have also been reported [9,10]. Our previous studies have shown that coarse and fine mineral particles induce differential responses in lung cells [11,12]. Furthermore, several *in vivo *and *in vitro *studies have demonstrated larger inflammatory responses to coarse fractions of PM compared to fine [13-15].

Various chemical components may influence the inflammatory potential of ambient particles. With respect to inorganic components, the importance of metals has been demonstrated. The ability of particles collected in the Utah Valley to induce cytokine production correlated with their metal content [16]. Transition metals in the soluble fraction of residual oil fly ash (ROFA) were found to be responsible for increased release of inflammatory cytokines [17,18]. However, such concentrations of metals are rather unusual and cannot explain health outcomes of all epidemiological studies. In other *in vitro *studies with rat lung macrophages, however, inflammatory responses to PM could not be explained by variations in the concentrations of soluble metals, but seemed attributable to the insoluble components of the particles [19,20]. Organic components of PM may also elicit inflammatory responses [21]. Microbial components bound to the particles, e.g. endotoxin, may contribute to the inflammatory potential of ambient PM. *In vitro *studies have demonstrated stronger pro-inflammatory effect of the coarse (2.5 – 10 μm) than the fine (< 2.5 μm) fractions of PM_10 _and attributed these effects to the endotoxin content, even though some of the endotoxin was found in the fine fractions [14,22-24].

Alveolar macrophages and different types of epithelial cells constitute the primary targets of inhaled lung toxicants and are therefore particularly important in the induction of inflammatory responses in the lung. Distinct particle properties involved in the activation of various uptake mechanisms or the triggering of responses by interactions between particles and receptors on the plasma membrane may in turn lead to different cytokine responses. Macrophages release a variety of inflammatory cytokines upon particle exposure, such as tumour necrosis factor (TNF)-α and interleukin (IL)-6 [25]. TNF-α is known to stimulate, among other effects, the epithelial cells to release various cytokines involved in the recruitment and activation of inflammatory cells, and also enhance the response to subsequent treatment with particles [26]. IL-6 serves as a chemoattractant for lymphocytes [27].

This work was performed within the scope of a European Union sponsored project entitled "Respiratory Allergy and Inflammation Due to Ambient Particles" (RAIAP). The overall objective was to assess the role of ambient suspended particles in causing inflammation in the respiratory tract and induction and elicitation of respiratory allergies. Representative samples of ambient particulate matter (PM_2.5–10 _and PM_0.1–2.5_) were collected in cities across Europe with expected differences in PM composition and traffic intensity (Amsterdam, Lodz, Oslo and Rome) during spring, summer and winter seasons. In the study presented here, we investigated the ability of the particle samples to induce release of the pro-inflammatory cytokines TNF-α and IL-6 from primary alveolar macrophages isolated from rats. Data from these *in vitro *studies and data from the particle characterisation studies [28] were then related in order to disclose possible relations between the potential to induce inflammatory markers and the presence of specific components of the various particle samples.

## Results and discussion

### Particle samples

Particles were sampled in four major cities (Rome, Amsterdam, Lodz and Oslo) representing the southern, western, eastern and northern part of Europe using a high-volume cascade impactor (HVCI) [29]. The cities were selected based on the expected differences in chemical composition rather than on known differences in health status due to PM exposure. In this light it should be mentioned that we did not aim to compare the cities explicitly. Both seasonal and regional differences in PM mass were observed during the collection period (2001 – 2002) [28]. Generally, the highest levels of PM were observed during winter (Lodz and Rome), whereas the lowest levels were observed in the spring (Oslo and Amsterdam). Variations in the relative contribution of pollution sources within, as well as between the cities, may be illustrated by variations in the observed ratio between fine and coarse particles collected during the different seasons (Table 1). In summer, the fine fraction constituted 50 – 60% of the total amount in each city based on the HVCI data. During winter, however, fine particles represented 77% of the total mass in Lodz compared to 51% in Oslo. This higher percentage of fine PM in the winter particulate air pollution in Lodz may be explained by the greatly increased use of fossil fuels for heating. In Oslo, a relatively low amount of fine particles compared to coarse was found during winter (51%) and spring (42%). This may partly be explained by the generation of mineral particles due to extensive road surface abrasion by cars with studded tires and sand sprinkling on icy roads. It should be mentioned that the technique of high-volume sampling is not ideal to determine actual ambient particle concentrations over time and these values might deviate from reference methods.

**Table 1:** Proportion of fine and coarse fraction of the collected PM estimated for each city and each season. The proportion is expressed as mass of the fine fraction in percent of the total mass (coarse + fine fraction) collected by high-volume cascade impactors in each sampling period.

	**Spring (percent fine of total)**	**Summer (percent fine of total)**	**Winter (percent fine of total)**
Lodz	58	54	77
Rome	53	52	68
Oslo	42	60	51
Amsterdam	55	60	56

### Cytokine release in relation to localization and season

**Figure 1:**
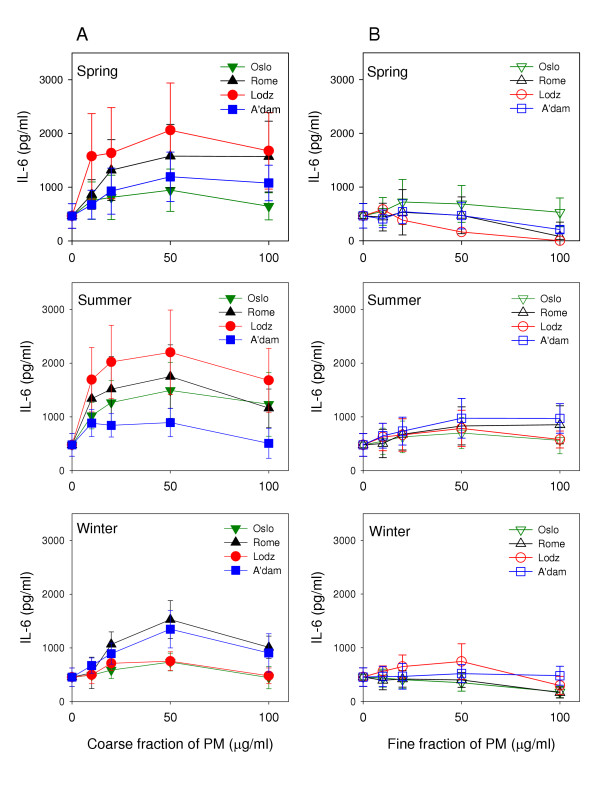
Release of IL-6 after exposure of alveolar rat macrophages to increasing concentrations of coarse (lane A) and fine (lane B) fractions of ambient PM collected in Oslo, Rome, Lodz and Amsterdam during spring (upper), summer (middle) and winter (lower) seasons. Values are mean ± SEM (n = 3).

Figure 1 displays the effect of increasing concentrations of PM on IL-6 release from alveolar macrophages. The coarse samples collected in Lodz during spring and summer appeared to be the most potent, reaching 440% and 460% increase, respectively, compared to control. The coarse summer fractions collected in Rome and Oslo induced higher levels of IL-6 than the corresponding sample from Amsterdam (370% and 310% versus 190% in maximal increase) (Figure 1, lane A). The coarse fractions of the winter season samples, however, exhibited a different order of potency compared to spring and summer season samples. Both the samples from Rome and Amsterdam induced higher levels of IL-6 than the samples from Lodz and Oslo (340% and 300% versus 165% and 160%, respectively). Generally the fine fractions did not induce any significant release of cytokines, even though a slight dose-dependent increase was observed after exposure to all fine summer samples and the fine winter sample from Lodz (Figure 1, lane B).

**Figure 2:**
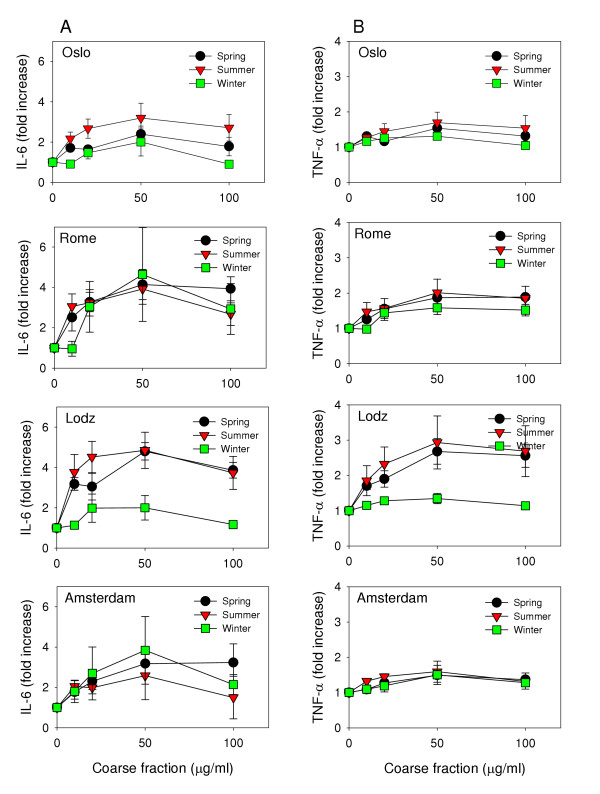
Seasonal variations in release of IL-6 (lane A) and TNF-α (lane B) from alveolar rat macrophages after exposure to the coarse fractions of ambient PM collected in Oslo, Rome, Lodz and Amsterdam (top to bottom). Values are shown as fold increase compared to control levels (IL-6_ctr_.: 455 – 477 pg/ml, SEM: 172 – 230; TNF-α_ctr._: 382 – 679 pg/ml, SEM: 13 – 23). Values are mean ± SEM (n = 3).

A distinct seasonal variation in potential to induce IL-6 was demonstrated among the coarse fractions collected in Lodz (Figure 2, lane A). A notably lower level of IL-6 was induced by the winter sample compared to the spring and summer samples. In contrast to the Lodz results, the coarse fractions collected during the different seasons in Rome seemed equally potent. The coarse summer fraction from Oslo induced a higher level of IL-6 release compared to the spring and winter samples, though not statistically significant. The Amsterdam coarse fraction collected during winter induced the highest level of IL-6, which is in contrast to the other three cities. However, the differences in Amsterdam did not reach statistical significance. In general, the highest pro-inflammatory potential seemed to be found in particles collected during spring and summer, the seasons with the highest prevalence of allergens.

Both particle fractions induced a relatively similar pattern of responses with TNF-α as with IL-6, although the responses in Oslo, Rome and Amsterdam were lower than for IL-6. The results from the coarse fractions from each city are presented as fold increase in Figure 2, lane B. The coarse fractions collected during spring in Rome and Lodz demonstrated a significant increase in TNF-α release compared to control. The fine fractions did not induce a marked increase in TNF-α release in any season or city (data not shown). The macrophages used in this study were from healthy animals. A different result may have been demonstrated with primed or activated macrophages. In vitro studies have demonstrated increased levels of PM-induced inflammatory responses in macrophages primed with LPS when compared to unprimed [26]. Furthermore, primed epithelial cells have also been shown to have augmented proinflammatory responses to particles [30]. This may imply that inflammatory potent particles could be even more potent to persons with preexisting inflammation.

### Cytokine release in relation to coarse and fine fractions

The cytokine release induced by the coarse fractions was notably higher compared to the cytokine release induced by the fine fractions (Figure 1). In a related study, in which rat type 2 cells were exposed to the coarse and fine fractions of the collected samples, the particle-induced release of MIP-2 demonstrated a relatively similar pattern of responses as in the macrophages [31]. These results are in accordance with other studies who report that the coarse fractions of ambient PM appear to be more potent than the fine fractions to induce inflammatory responses [14,20,24,32]. The authors attributed the greater inflammatory potential of the coarse particles to higher levels of bioactive biological components bound primarily to the coarse fraction. In a study in mice, however, inflammatory responses after exposure to fine and ultrafine fractions of ambient particles were similar or even greater compared to coarse [13]. The very low responses after exposure to the fine fractions observed in our study could be influenced by a certain loss of ultrafine particles due to the mode of operation of the high volume sampler [29,33]. However, we exposed A549 cells to the similarly collected fine particle samples as used with the macrophages and a dose-dependent increase in IL-8 was observed (unpublished results). Similar levels of IL-8 release were also demonstrated when A549 cells were exposed to low concentrations of coarse, fine and ultrafine fractions of urban particles collected with a similar sampler as used in this study [34]. The previous explanation appears therefore less likely. It has, however, been shown that diesel particles may have a suppressive effect on the cytokine release from alveolar macrophages [35]. Thus, if diesel particles constitute a considerable part of the fine particle fractions in our study, a corresponding suppression might be an explanation.

### Cytokine release in relation to chemical components

Major differences in chemical composition were observed in the collected RAIAP-samples [28]. The predominance of combustion particles in Lodz is reflected in a high content of zinc, PAH and other organic components compared to the other samples. In contrast, ambient PM in Rome seems to be influenced by long distance transboundary particles from the African continent, resulting in relatively high levels of metals and mineral components. Amsterdam is close to the North Sea, and the ambient PM will therefore contain sea salt, as well as long distance transported particles. Typical for Oslo, especially in winter, is a relatively high level of PAH in addition to inorganic components. The PAH is most likely a result of extensive wood burning for heating. Temporal and spatial variation in chemical composition of ambient PM and a corresponding variation in biological activity have previously been reported by other investigators [20,36-39].

#### Elements

**Figure 3:**
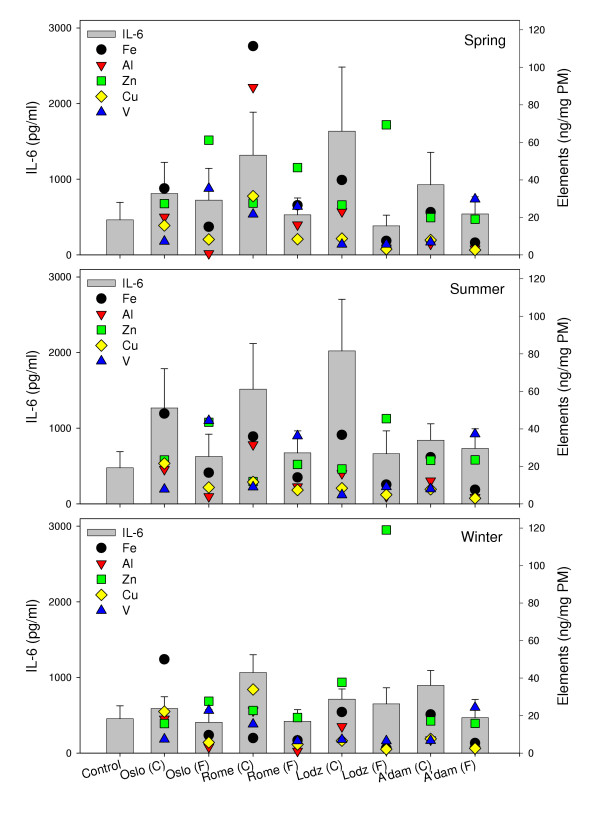
The concentrations of selected elements in the PM samples presented together with the respective PM-induced IL-6 from alveolar rat macrophages after exposure to 20 μg/ml of coarse (C) and fine (F) fraction collected in Oslo, Rome, Lodz and Amsterdam in spring (upper), summer (middle) and winter (lower) seasons. IL-6 release is shown on the left ordinate, values in diagram are mean ± SEM (n = 3). Concentration of elements is shown on the right ordinate (ng/mg PM). Indicated values in the diagram for Fe and Al should be multiplied by 10^3^, values for Zn and Cu by 10^2 ^and values for V by 10^1^.

The elements iron (Fe), aluminium (Al) and copper (Cu) are typical for both crustal material and other sources, whereas zinc (Zn) and vanadium (V) are elements more related to various combustion processes. The relationship between the content of selected elements and release of IL-6 after exposure of the macrophages to non-toxic levels of particles (20 μg/ml) is presented in Figure 3. The coarse fractions were characterised by a higher content of Fe, and to a certain extent also Cu and Al, than their respective fine fractions. Higher levels of these metals may therefore be related to the higher cytokine responses induced by the coarse particles. In contrast, the fine fractions, which induced no or only small cytokine responses, seemed to contain the highest levels of Zn and V. Especially, the fine winter sample collected in Lodz stands out with a much higher content of Zn than any of the other samples.

Several studies have focused on an importance for redox-active transition metals such as Fe and Cu in lung inflammatory responses, partly due to their ability to participate in Fenton chemistry and the production of reactive oxygen species (ROS) [40]. Furthermore, both V and Zn have been suggested to contribute to inflammatory reactions through their effect as inhibitors of protein phosphatases [41]. Aluminium salts, on the other side, have been associated with a decreased inflammatory potential of particles (quartz) by modifying the particle surface [42,43]. With respect to the importance of metals for PM-induced responses, an effect of both soluble and insoluble fractions has been reported [17-19,44,45]. This supports that the metal content may contribute to the observed higher levels of cytokines induced by the coarse fractions in our study. Apparently, the concentrations of V and Zn found in our samples are not high enough to induce any cytokine release in the alveolar macrophages. An exception might be a relation between the high content of Zn in the fine winter fraction from Lodz and the slight dose-dependent increase in IL-6 release (Figure 1, lane B). Another aspect in this study is that the cytokine responses are compared to the total amounts of metals in the particle samples and not the soluble fractions.

Although Fe, Cu and Al seemed to be prevalent in the coarse particle samples and might contribute to the cytokine-releasing potential of the coarse versus the fine fractions, the occurrence of any single element was not sufficient to explain the regional or seasonal heterogeneity between the coarse particles. This is illustrated by the fact that the cytokine release induced by the coarse spring sample from Lodz, in which the content of Fe was relatively low, equalled the response induced by the coarse samples from Rome, the most Fe-rich samples of all. The potential of the different particle samples may therefore be influenced by combinations of various elements or components. An increased inflammatory response has been shown after exposure to combinations of Cu/Zn compared to exposure to the single elements [46].

#### PAH

**Figure 4:**
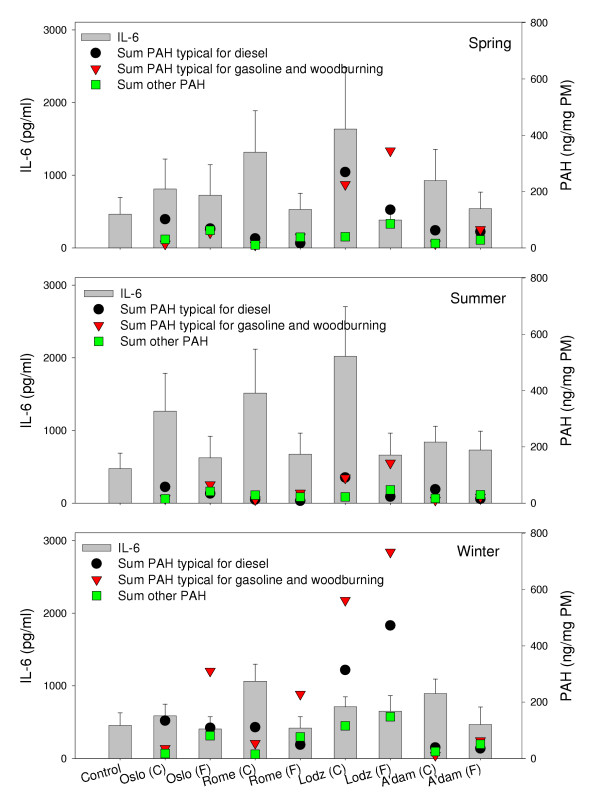
The concentrations of subgroups of PAH in the collected samples presented together with the respective PM-induced IL-6 from alveolar rat macrophages after exposure to 20 μg/ml of coarse (C) and fine (F) fraction collected in Oslo, Rome, Lodz and Amsterdam in spring (upper), summer (middle) and winter (lower) seasons. IL-6 release is shown on the left ordinate, values in the diagram are mean ± SEM (n = 3). Concentration of PAH are shown on the right ordinate (ng/mg PM). Values of the subgroup of PAH representing emissions from combustion of diesel are the summarised concentrations of phenantrene, 1-methylphenantrene, fluoranthene and pyrene in each PM-sample. Values of the subgroup of PAH representing emissions from gasoline engines and wood burning are the summarised concentrations of naphthalene, benz[*a*]anthracene, chrysene, benzo[*b*]fluoranthene, benzo[*k*]fluoranthene and benzo[*g,h,i*]perylene in each sample. Values of other PAH represent the sum of acenaphthene and benzo[*a*]pyrene in each sample.

A wide range of PAH is associated with PM derived from combustion of materials such as diesel, gasoline, coal and wood. The different size fractions of ambient PM may contain various amounts of these compounds. In Figure 4, the content of subgroups of PAH associated with particles resulting from combustion of carbonaceous material is presented in relation to the observed IL-6 responses. The coarse fractions contained equal or higher levels of some PAH typically found in emission from combustion processes dominated by diesel. One exception was the very high level of these PAH found in the fine winter fraction collected in Lodz. Moreover, the fine fractions demonstrated equal or higher concentration of the other two subgroups of PAH (PAH from gasoline and woodburning and other PAH) than the respective coarse fractions. However, variations in neither the total PAH content nor the PAH content of the subgroups could explain the higher potency of the coarse fractions, or the observed seasonal and geographical differences in IL-6 release. This is best illustrated by the samples from Lodz and Rome, in which the coarse fractions collected in summer, with relatively low PAH content, induced the highest cytokine release. In contrast, exposure to the most PAH rich samples, the coarse and fine winter fractions from Lodz, induced much lower levels of IL-6. It has been shown that organic compounds that induced CYP1A1 expression were critical for the inflammatory response induced by diesel exhaust in airway epithelial cells [21]. In our studies, it should not be excluded, however, that the apparent lack of association between cytokine release and PAH content might be due to low metabolism of PAH to reactive metabolites in the alveolar macrophages studied. This notion is supported by a recent study, in which CYP1A1 protein levels were found very low, and hence the toxicity of the PAH minimal in alveolar rat macrophages [47]. Furthermore, another study indicated that the total soluble organic fractions, rather than specific PAH, were involved in the inflammatory responses [48].

#### Inorganics

**Figure 5:**
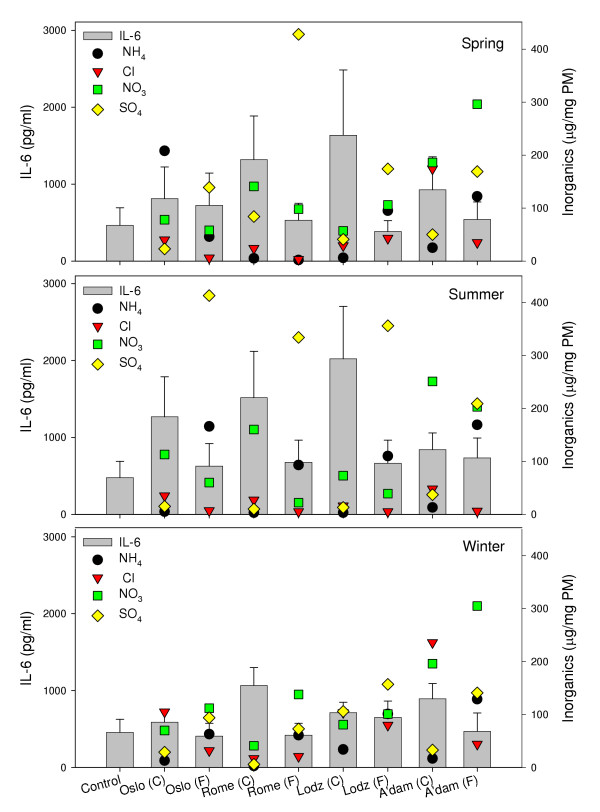
The concentrations of selected inorganic components in the collected samples presented together with the PM-induced IL-6 from alveolar rat macrophages after exposure to 20 μg/ml of coarse (C) and fine (F) fractions collected in Oslo, Rome, Lodz and Amsterdam in spring (upper), summer (middle) and winter (lower) seasons. IL-6 is shown on the left ordinate, values are mean ± SEM (n = 3). The concentration of inorganics is shown on the right ordinate (μg/mg PM).

In Figure 5, the relative amounts of some inorganic compounds in relation to the particle-induced release of IL-6 are shown. The coarse fractions contained equal or higher levels of chloride compared to the fine fractions, in which higher levels of ammonium and sulphate were demonstrated. The various levels of inorganic components measured in the PM samples did not seem to be associated with the higher levels of cytokine release induced by the coarse fractions. Furthermore, no relation was revealed between seasonal and geographical variations and content of the inorganics. This is in accordance with conclusions from the current toxicological database regarding health effects and inorganic components of ambient air particles, which concluded that these components had a low potential for health effects [49]. The observed high levels of sulphate in the fine summer samples may in part be due to more warm and humid conditions favourable for more rapid oxidation of sulphur dioxide generated from combustion of fossil fuels.

#### Endotoxin

**Figure 6:**
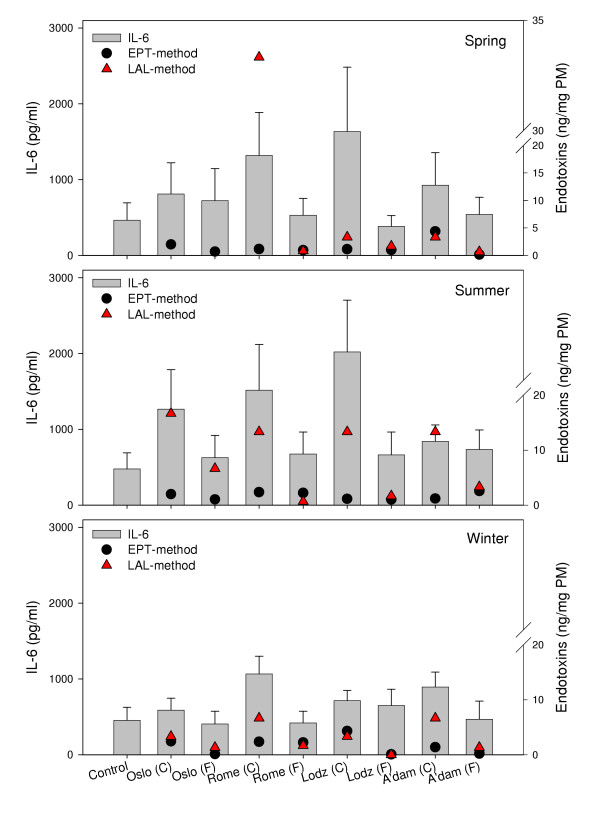
The concentrations of endotoxins in the collected samples presented together with PM-induced IL-6 from alveolar rat macrophages after exposure to 20 μg/ml of coarse (C) and fine (F) fractions collected in Oslo, Rome, Lodz and Amsterdam in spring (upper), summer (middle) and winter (lower) seasons. IL-6 release is shown on the left ordinate, values in diagram are mean ± SEM (n = 3). Concentration of endotoxin is shown on the right ordinate (ng/mg PM). Endotoxin content in PM samples measured by the LAL-method and the EPT-method are shown. LAL-analysis of the Oslo spring samples is missing due to shortage of sample material.

In Figure 6, the endotoxin levels measured by the Limulus amoebocyte lysate (LAL)-test and the Endotoxin Pyrogen Test (EPT) are presented in relation to the particle-induced release of IL-6. The coarse fractions showed a higher content of endotoxin than the fine fractions when related to the results from the LAL-test. This is in accordance with several other studies [14,20,22,24,32]. Also, the coarse summer samples from Oslo, Lodz and Amsterdam contained higher levels of endotoxin than the spring and winter samples when analysed by this method. A higher level of endotoxin in particles collected during the warmer season has also been reported by others [50]. Thus, there may be a trend in our study that the observed higher IL-6 levels induced by the coarse fractions are associated with higher endotoxin levels. However, there is no direct proportionality, as illustrated by the differing results from Lodz and Amsterdam. Spring and summer particles from Lodz induced notably higher cytokine release compared to the particles from Amsterdam, whereas the particles from Amsterdam contained similar or higher levels of endotoxin. The notion that the presence of endotoxin may contribute to the observed higher potential of the coarse fractions than the fine, is to a certain extent supported when endotoxin was analysed by the more quantitative EPT-assay. Higher levels of endotoxin in the coarse than the fine samples were found. Compared to the LA assay, consistently lower levels, as well as less variation between samples were measured by this method. Furthermore, no relation between the seasonal or site-specific variation in cytokine release and the EPT-results on endotoxin content was observed. The discrepancy in endotoxin content measured by these methods may be explained by an overestimation of the levels in the LAL-assay. Other organic components, such as β-glucans, were detected (but not quantified) in PM from all seasons and cities [51]. The presence of these components may have influenced the LAL-result, since glucans, proteins and other agents have demonstrated a capability to activate the LAL-assay [52].

**Figure 7:**
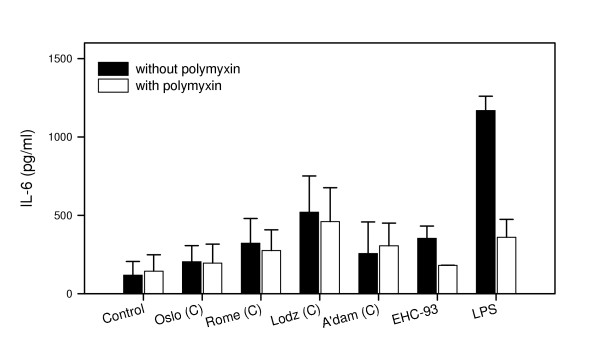
Release of IL-6 from alveolar rat macrophages after exposure to 20 μg/ml of the coarse (C) PM samples collected during the summer season, EHC-93 or LPS with or without treatment of polymyxin B sulphate. Values are mean ± SEM (n = 3).

To further study the involvement of endotoxin, the particle samples were treated with the endotoxin-binding protein polymyxin B sulphate (polymyxin) before addition to the cell cultures. Lipopolysaccaride (LPS), an endotoxin from Gram negative bacteria, was included as a positive control. The LPS-binding property of polymyxin was demonstrated by the complete reduction in IL-6 release induced by LPS and a marked reduction in IL-6 induced by the urban standard PM EHC-93 (Figure 7). However, no significant reduction in the cytokine release was observed after polymyxin-treatment of the collected particle samples. This indicated that the observed differences in potential of the coarse fractions to induce IL-6 release could not be attributed to their content of endotoxin. Synergistic interactions have also been suggested for endotoxin and other proinflammatory components [53]. Such possibilities, as well as a role of other constituents of biological origin cannot be excluded for the observed results in our study.

In summary, the present studies demonstrated that ambient air particles collected in a western, an eastern, a northern and a southern European city differed in their potential to induce pro-inflammatory cytokines in primary macrophages isolated from rat lung. On a gram-by-gram basis significant differences in the levels of IL-6 and TNF-α release were observed after exposure to particle samples collected in the different cities and different seasons. The coarse fractions induced significant higher levels of cytokines than the fine fractions. Though specific metals and endotoxin seem to be prevalent in the coarse particle samples, the observed variations in potency were not related to variations in concentrations of any component in the particle samples. Interaction(s) between constituents in the different particle samples might be a probable explanation for the observed effects. Both interactions between different metals and metals and organic compounds are conceivable. The strongest cytokine responses were also observed of particles collected during spring and summer, the seasons with the highest prevalence of allergens. Given that a preceding or side-by-side non-specific PM-induced inflammation in the airways might influence the susceptibility of persons with respiratory allergies, seasonal heterogeneity in inflammatory potential of ambient PM may influence the onset or severity of allergic responses in the respiratory system.

## Conclusion

The cytokine-inducing potential of collected ambient PM varied between sampling-site and -season. Clear differences were also observed between the coarse and the fine PM fractions: the coarse fractions were consistently more potent than the corresponding fine fractions. A simple relationship between the presence of a specific component in the particle samples and their potential to induce cytokines could not be demonstrated.

## Methods

### Chemicals

Lipopolysaccharide (LPS) and polymyxin B sulphate were obtained from Sigma-Aldrich, St. Louis, MO, USA. Foetal bovine serum (FBS) was obtained from Gibco BRL, Paisley, Scotland. The antibiotics ampicillin and fungizone were purchased from Bristol-Myers Squibb AB, Denmark; penicillin/streptomycin and the culture medium RPMI 1640 were from BIO Whittaker, Walkersville, MD, USA. The enzyme-linked immunosorbent assays (ELISA) for analysis of rat TNF-α and IL-6 were obtained from R&D Systems Europe, Oxon, UK. Ottawa dust (EHC-93) was kindly provided by Dr. Renauld Vincent, Environmental Health Directorate, Health Canada, Ottawa, Ontario, Canada.

### Particle sampling

The PM sampling campaign is described in detail in the final project report [49]. In short, ambient air particles were collected in the cities Amsterdam, Lodz, Rome and Oslo during spring, summer and winter 2001/2002. A high-volume cascade impactor with a multi-stage slit nozzle impactor has been used to collect coarse (PM_2.5–10_) and fine (PM_0.1–2.5_) fractions on polyurethane foam (PUF) by impaction. The fractions of dry particle samples extracted with methanol from the PUFs were provided for the particle characterisation and biological experiments [28].

### Particle characterisation

Characterisation of the collected samples was performed at the National Institute for Public Health and the Environment (RIVM), the Netherlands, and is described in detail by Cassee et al. [28].

#### Elements and other inorganics

In summary, elemental composition of the particles was analysed with ICPMS. Secondary aerosols were detected with ion-chromatography (Cl, NO_3 _and SO_4_) or photometry (NH_4_), and anions were analysed using a Dionex guard column (AG-4A), Separation column (Dionex AS-A4) and pulsed electrochemical detector (Dionex-PED). The highest content of Fe was found in the coarse fractions when the coarse and fine fractions within each city were compared. The content of Cu in the coarse fractions generally exceeded the content in the fine fractions. In contrast, the highest levels of Zn and V were found in the fine fractions [28].

#### PAH

PAHs were analysed on a 30 m 0.25 mm WCOT DB-5MS column in a Fisons 8000 series gas chromatograph equipped with an Interscience MD800 mass spectrometer with EI in the SIR mode. The coarse fractions contained equal or higher levels of PAH typically found in emissions from combustion processes dominated by diesel fuelled engines (phenantrene, 1-methylphenantrene, fluoranthene and pyrene) compared to the fine fractions. The fine fractions demonstrated an equal or higher concentration of PAH typically generated by combustion of gasoline, as well as from wood burning (naphthalene, benz[*a*]anthracene, chrysene, benzo[*b*]fluoranthene, benzo[*k*]fluoranthene and benzo[*g,h,i*]perylene) and also other PAH (acenaphthene and benzo[*a*]pyrene) when compared to the coarse fractions.

#### Inorganics

The highest level of NH_4 _was found in the coarse sample collected in Oslo during spring. In all other samples, NH_4 _was higher in the fine than in the coarse fractions. A higher level in the fine compared to the coarse fractions was also observed for SO_4_. Cl was equal or higher in the coarse fractions, whereas the highest levels of NO_3 _were found in the fine fractions.

#### Endotoxins

Endotoxin concentrations were determined using the Limulus amoebocyte lysate (LAL) test (Limusate, Sigma-Aldrich Chemie BV, Zwinrecht, The Netherlands), as described by the manufacturer (detection level < 0.125 ng endotoxin/ml). Endotoxin content in the PM suspensions was also determined using the U.S. Pharmacopeia (USP) Endotoxin Pyrogen Test (EPT) (USP 23 NF 18, 1994, U.S. Pharmacopeial Convention, INC., Rockville, MD, USA). Generally, higher levels of endotoxin were found in the coarse fractions compared to the fine fractions within each city.

### Preparation of particles for biological studies

The collected dry particle samples were suspended in 0.9% NaCl to a concentration of 20 mg/ml and stirred overnight. The suspensions were further diluted in culture medium to stock solutions of 2 mg/ml. Before use in the exposure studies, the stock solutions were stirred on a magnetic stirrer overnight. Dry particle samples, as well as particle suspensions, were stored at -20°C. In experiments aimed to study if endotoxins were involved in the observed responses, stock solutions of particles were treated with the LPS-binding polymyxin B sulphate (10 μg/ml) for 1 hour before addition to the cells.

### Primary rat alveolar macrophages

Male rats (Crl/Wky) were purchased from Harlan, UK. Alveolar macrophages were collected by airway lavage, suspended in RPMI medium with supplements and added to 35 mm 6 well culture dishes (1.5 × 10^6^/well). Non-attached cells were removed after 1 hour, whereas the attached macrophages were used for exposure.

### Exposure of cell cultures to particles

Cells were cultured in FBS-free medium from the start of exposure and the subsequent 6 hours, then 5% FBS was added. After 20 hours of exposure in a total of 1 ml/well of culture medium, the medium was collected and centrifuged for 10 minutes to remove cells (250 × g) and to remove particles (2500 × g). Supernatants were stored at -70°C until further analysis of inflammatory cytokines. Responses after exposure to coarse and fine fractions collected within each season were studied in the same experiment and repeated at least three times.

### Cytokine assays

Analysis of the inflammatory cytokines (IL-6 and TNF-α) was performed using enzyme-linked immunosorbent assay (ELISA) according to the manufacturer's manual. The increase in colour intensity was quantified using a plate reader with software (TECAN Sunrise with Magellan V 1.10, Tecan Austria, Salzburg, Austria).

### Statistical analysis

The data were analysed for significance by One Way Analysis of Variance (ANOVA) (Tukey Test). Kruskal Wallis ANOVA on Ranks was used when Normality or Equal Variance Test failed (Dunn's Method). P < 0.05 was judged to be statistically significant.

## Competing interests

The author(s) declare that they have no competing interests.

## Authors' contributions

RBH performed the experimental studies, the biochemical and statistical analysis, and prepared the manuscript. ML, MR and PES performed the isolation of the macrophages and contributed to the writing of the manuscript. FRC was responsible for collection and physical-chemical characterisation of the particulate samples and contributed to the writing of the manuscript. ED coordinated the RAIAP project. All authors have read, reviewed, commented and approved the final manuscript.
